# Biological and environmental covariates of juvenile sockeye salmon distribution and abundance in the southeastern Bering Sea, 2002–2018

**DOI:** 10.1002/ece3.11195

**Published:** 2024-04-08

**Authors:** Ellen M. Yasumiishi, Curry J. Cunningham, Ed V. Farley, Lisa B. Eisner, Wesley W. Strasburger, John A. Dimond, Paul Irvin

**Affiliations:** ^1^ Auke Bay Laboratories, Alaska Fisheries Science Center, Ted Stevens Marine Research Institute NOAA Fisheries Juneau Alaska USA; ^2^ College of Fisheries and Ocean Sciences University of Alaska Fairbanks Juneau Alaska USA; ^3^ Alaska Fisheries Science Center NOAA Fisheries Seattle Washington USA

**Keywords:** abundance, climate, distribution, ecosystem, marine, salmon

## Abstract

Climate change is altering the distribution and abundance of marine species, especially in Arctic and sub‐Arctic regions. In the eastern Bering Sea, home of the world's largest run of sockeye salmon (*Oncorhynchus nerka*), juvenile sockeye salmon abundance has increased and their migration path shifted north with warming, 2002–2018. The reasons for these changes are poorly understood. For these sockeye salmon, we quantify environmental and biological covariate effects within spatio‐temporal species distribution models. Spatio‐temporally, with respect to juvenile sockeye salmon densities: (1) sea surface temperature had a nonlinear effect, (2) large copepod, *Calanus*, a minor prey item, had no effect, (3) age‐0 pollock (*Gadus chalcogrammus*), a major prey item during warm years, had a positive linear effect, and (4) juvenile pink salmon (*O*. *gorbuscha*) had a positive linear effect. Temporally, annual biomass of juvenile sockeye salmon was nonlinearly related to sea temperature and positively related to age‐0 pollock and juvenile pink salmon abundance. Results indicate that sockeye salmon distributed with and increased in abundance with increases in prey, and reached a threshold for optimal temperatures in the eastern Bering Sea. Changes in population dynamics and distribution of sockeye salmon in response to environmental variability have potential implications for projecting specific future food securities and management of fisheries in Arctic waters.

## Graphical abstract

This manuscript describes changes in the distribution and abundance of juvenile sockeye salmon from Bristol Bay, the world's largest run of sockeye salmon, in relation to spatio‐temporal and temporal changes in prey, competitors, and temperature. Spatio‐temporally varying densities of juvenile sockeye salmon varied with sea temperature, age‐0 pollock and juvenile pink salmon, but not *Calanus* copepods. Temporally, increases in annual biomass of juvenile sockeye salmon corresponded nonlinearly with warming, positively with age‐0 pollock (a highly abundant prey during warm years), positively with juvenile pink salmon (a potential competitor), but did not correspond with large copepods (smaller prey).

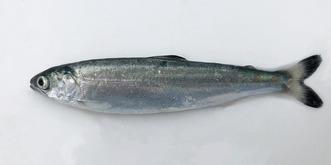

## 1 INTRODUCTION

Climate change is dramatically altering the distribution and abundance of marine species (Campana et al., 2020; Hunt Jr et al., 2018; Perry et al., 2005; Yasumiishi et al., 2020). Polar regions are experiencing faster rates of ecosystem change than temperate and tropical regions (You et al., 2021). In Arctic and sub‐Arctic regions, warming has increased atmospheric and ocean temperatures, precipitation, and river discharge that increases nutrient delivery to nearshore waters and has reduced snow cover and winter sea ice that impacts freshwater and saltwater habitats (Box et al., 2019; Hermann et al., 2019). Regional Ocean Modeling System forecasts predict: (1) upward trends in downward longwave radiation, air temperature, absolute humidity, sea surface temperature, sea bottom temperature, sea surface height, and cross‐shelf transport, and (2) downward trends in mixed layer depth (more negative values indicate mixed layer deepening), ice cover, sea surface salinity, nutrients, benthic and epibenthic detritus, ice‐associated primary production, phytoplankton, copepods, and euphausiids for the eastern Bering Sea (EBS) through 2100 (Hermann et al., 2019). These changes impact marine species distribution and abundance, ecosystem food webs, and the community composition of phytoplankton, zooplankton, fish, jellyfish, birds, and marine mammals (Sigler et al., 2011). Currently, implications of warming on subsistence, sport, and commercial fisheries as well as the culture and well‐being of western Alaskan communities include increases in sockeye salmon (*Oncorhynchus nerka*), chum salmon (*O*. *keta*), pink salmon (*O*. *gorbuscha*), sablefish (*Anoplopoma fimbria*), Pacific Ocean Perch (*Sebastes alutus*), and walleye pollock (*Gadus chalcogrammus*) stocks and reductions in Pacific cod (*Gadus macrocephalus*), and Chinook salmon (*O*. *tshawytscha*) stocks. Changes in the relative abundance of these stocks have co‐occurred with changes in ocean temperatures. We assume that future warming will continue these downward trajectories, while upward‐trending species will reach a threshold under extreme warm conditions.

The sub‐Arctic EBS has experienced large‐scale ecosystem variation in physical and biological properties during recent warm (2002–2005), cool (2006–2012), and warm (2013–2018) stanzas (Coyle et al., 2011; Hunt Jr et al., 2011, 2020; Sigler et al., 2011). In the EBS, cool stanzas are often described as multiyear periods exhibiting a greater extent of spring sea ice followed by cooler summer sea temperatures, higher densities of large copepods and euphausiids on the shelf, and a current that flows northward during winter and more variable flow during other seasons. Conversely, warm stanzas represent periods of less sea ice during spring followed by warmer summer sea temperatures, lower densities of large copepods and euphausiids, and a current that flows westward from the shelf to oceanic areas (Stabeno et al., 2012).

The EBS is a shallow productive shelf region that lies between the Aleutian Islands and Bering Strait, a corridor to the Chukchi Sea in the Arctic Ocean, where sea temperature and ice are important drivers of ecosystem change (Coyle et al., 2011; Eisner, Yasumiishi, et al., 2020; Eisner, Zuenko, et al., 2020; Hunt Jr et al., 2011; Yasumiishi et al., 2019). As sea ice forms, cold water sinks to the benthos and acts as a thermal barrier for demersal species and a refuge for pelagic fishes, called the cold pool. Ice‐associated phytoplankton also provides food for zooplankton production and growth (Durbin & Casas, 2013). In the EBS, a warming induced reduction of sea ice, and the cold pool we associated with cascading ecological effects, including (1) decreases in large copepod abundance (a lipid‐rich prey for fish), decreases in juvenile growth rates of Chinook salmon and lower capelin (*Mallotus villosus*) abundance, (2) increases in growth rates for juvenile sockeye salmon, and increases in the abundance of herring (*Clupea pallasii*), age‐0 pollock, and juvenile sockeye salmon, and (3) movement of juvenile salmon north into Arctic waters (Andrews et al., 2016; Farley Jr et al., 2020; Yasumiishi et al., 2020). The impacts of temperature and sea‐ice‐related changes in habitat, prey quantity and quality, predators, and competitors vary depending upon the species.

Many commercial, subsistence, and sport fish species have begun to move northward with warming in the EBS, both during their juvenile and adult life stages (Barbeaux & Hollowed, 2018; Eisner, Zuenko, et al., 2020; Hollowed et al., 2012; Rooper et al., 2021; Stevenson & Lauth, 2019; Thorson, 2019a; Yasumiishi et al., 2020). Northward shifts in distribution may be the result of species seeking thermal preferences, tracking changes in prey distribution, and/or avoiding predators and competitors. Species distribution models are increasingly used as a tool for understanding how fish species respond to physical and ecological covariates that change across time and space (Rooper et al., 2021; Thorson, 2019a).

The world's largest run of sockeye salmon originates from Bristol Bay river systems that flow into the sub‐Arctic waters of the EBS. Relative to the long‐term average run of 35.1 million Bristol Bay sockeye salmon, recent returns reached a record of 62.3 million fish in 2018, followed by large runs of >50 million fish in 2019 and 2020 (Brenner et al., 2020). Recent warming trends corresponded with declines in the body size of adult sockeye salmon but increases in the total biomass of commercial harvest and escapement (Oke et al., 2020). Reductions in body size can result in reduced fitness, fecundity, and genetic diversity but also lower size‐selective fishing and natural mortality (Cunningham et al., 2013; Darwin, 1874; Kendall et al., 2009). Understanding the direct and indirect influences of climate and ecosystem change on the distribution and abundance of marine fish species is key to understanding their vulnerability, survival, and ability to adapt. This knowledge is required to increase the accuracy of predictions for future change (Spencer et al., 2019) and provide a necessary foundation for climate‐adaptive fishery management policies.

Pelagic waters of the EBS serve as an important rearing habitat for these juvenile sockeye salmon during their first summer at sea, a time thought critical to their overwintering survival (Farley, Murphy, Adkison, Eisner, & Helle, et al., 2007; Farley Jr, Murphy, Adkison, & Eisner, et al., 2007). For juvenile sockeye salmon in the EBS, warming has been associated with a more westerly and northerly distribution, age‐0 pollock as a primary prey item, increases in biomass, higher growth rate potential, and higher energy status since the early 2000s (Coyle et al., 2011; Farley et al., 2011; Yasumiishi et al., 2020). The energy density of juvenile sockeye salmon is important in determining their overwintering survival in the EBS (Farley et al., 2011) and is driven in part by temperature, density‐dependent processes, and prey quality and quantity (Farley et al., 2011; Heintz et al., 2013). Therefore, it is important to understand how climate‐related ecosystem change drives the returns of adult sockeye salmon to Bristol Bay rivers entering the EBS, a species facing warming conditions at the northern edge of their global distribution.

**FIGURE 1:**
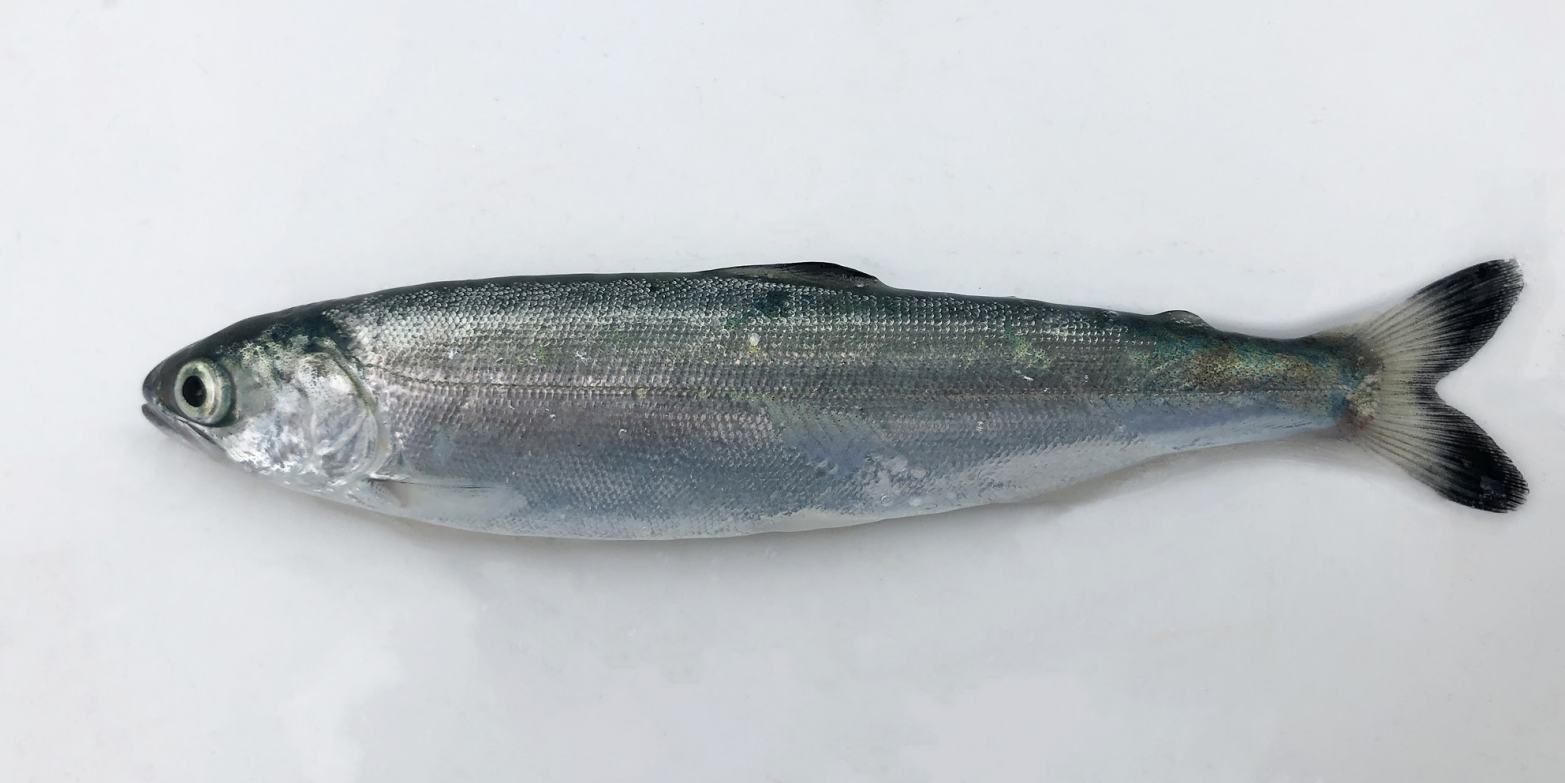
Juvenile sockeye salmon (*Oncorhynchus nerka*) captured during the first year at sea (Credit: Steve Heinl, Alaska Department of Fish and Game).

**FIGURE 2:**
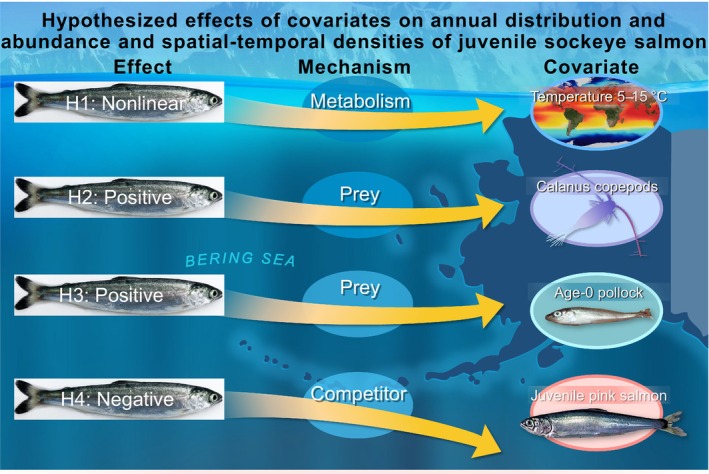
Conceptual model for the hypothesized effects of environmental and biological covariates on the distribution, abundance, and densities of juvenile sockeye salmon in the eastern Bering Sea. Direction of the arrow is upward for a positive or nonlinear (for temperature), and downward for a negative association between covariates and the distribution and abundance of juvenile sockeye salmon.

In this study, we explored temporal (inter‐annual) and spatio‐temporal (intra‐annual) changes in the distribution and abundance of juvenile sockeye salmon (Figure 1) in the EBS over 17 years and how these changes relate to 5–14°C sea temperatures (i.e., warm vs. cool, optimal ranges 7–15°C for juvenile sockeye distribution) (Echave et al., 2012), prey (i.e., age‐0 pollock vs. *Calanus*), and competitors for zooplankton forage (i.e., juvenile pink salmon) during late summer. We hypothesized (Figure 2) that the temporal trends in abundance and distribution (i.e., northward, westward, and expanded ranges) and spatio‐temporally varying densities of juvenile sockeye salmon in the EBS marine environment are positively associated with: (1) optimum water temperatures to maximize growth rate potential and maximize prey availability to attain higher energy status (Farley Jr, Murphy, Adkison, & Eisner, et al., 2007), (2) a primary zooplankton group (*Calanus* spp., hereafter *Calanus*) that is a key prey item of age‐0 pollock and a minor prey item of juvenile sockeye salmon (Coyle et al., 2011), and (3) abundance of a primary prey such as age‐0 pollock. Conversely, we hypothesized a negative association between juvenile sockeye salmon competitors such as juvenile pink salmon that also consume zooplankton and age‐0 pollock. In our study, temporal analyses provide an indication of possible ecological drivers of the distribution and abundance of juvenile sockeye salmon. Additional spatio‐temporal analyses provide more insight into where these ecological interactions occur and how interactions vary in space among years for a snapshot of the season. Studying a period of sequential warm‐cool‐warm stanzas provides insight into the impact of changing temperature and its effect on downstream ecosystem factors (i.e., prey items and competitors) influencing the distribution and abundance of juvenile sockeye salmon.

## 2 METHODS

### 2.1 Study area

**FIGURE 3:**
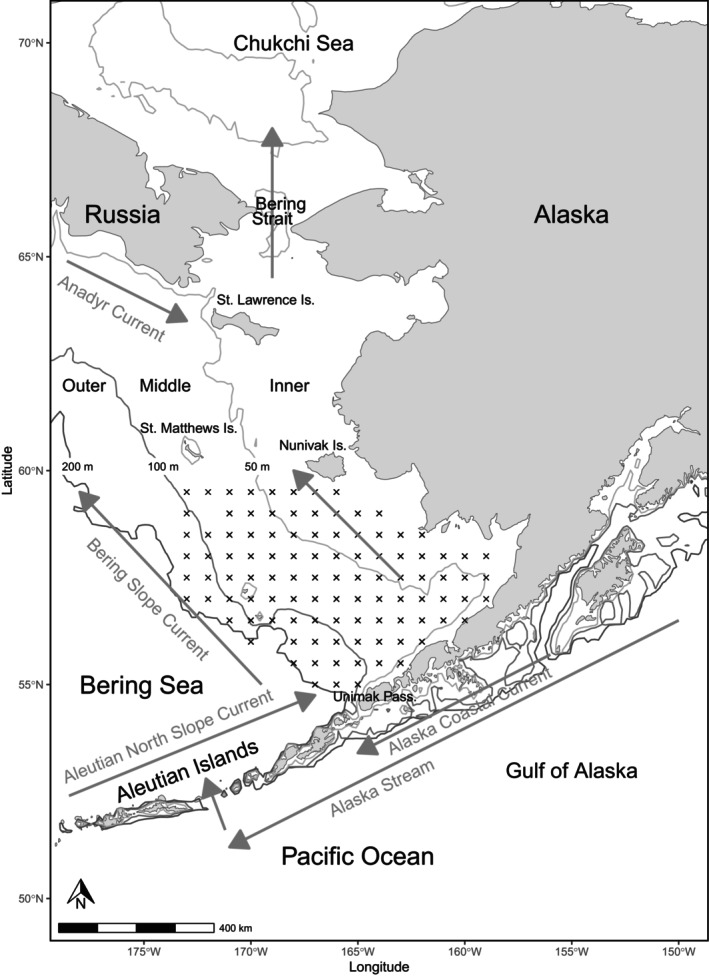
Map of the study area of the southeastern Bering Sea with symbols (x) showing survey locations among all years. Not all stations were surveyed each year. Arrows indicate dominant ocean current patterns that structure marine ecosystem dynamics within this region.

The EBS is an important rearing habitat for the juvenile sockeye salmon in our study (Echave et al., 2012). The EBS is bounded by the Aleutian Island Chain in the south and the Bering Strait in the north (Figure 3). Current movement into the south EBS from the Gulf of Alaska enters via multiple pathways. The majority of eastward flow above the EBS shelf originates from Unimak Pass, turning east north of the Pribilof Islands, and via the Anadyr Current south of St. Lawrence Island. Northward flow out of the EBS occurs through the Bering Strait, entering the Chukchi Sea. Shelf bathymetry is typically separated into three oceanographic domains, defined by their bathymetry and physical characteristics (Coachman, 1986). The inner domain is nearshore (<50 m bathymetry), weakly stratified, and consists of nutrient‐poor coastal waters (Kachel et al., 2002). The middle domain (50–100 m bathymetry) is a highly stratified two‐layer system in summer, with surface nutrients typically depleted except during storm events when there is episodic injection of nutrients from deep waters (Eisner et al., 2016). The outer domain (100–180 m) has a 3‐layer system with gradually stratified surface and deep waters, a well‐mixed middle layer, and moderate surface nutrients during the summer (Eisner et al., 2016). The narrow shelf break (~180–200 m) is defined as the “Green Belt” due to the higher nutrients and phytoplankton biomass driven by upwelling at the shelf edge (Springer et al., 1996). The EBS survey area is from the nearshore inner shelf to the shelf break, latitude 55° N to 59.5° N, and longitude 173° W to 159° W (Figure 3). The broad, shallow continental shelf has few geographic barriers and allows for fish movement north–south and east–west. Juvenile sockeye salmon rearing within the EBS are primarily outmigrants from the freshwater rivers of Bristol Bay, Alaska, spending the summer in the middle domain feeding, then migrating offshore and south of the Aleutian Islands into the central Pacific Ocean and Gulf of Alaska until maturing after 1–3 years at sea, when they return to Bristol Bay rivers to spawn.

### 2.2 Data

#### 2.2.1 Survey

Fish, zooplankton, and temperature information was collected in the EBS south of Nunivak Island during late summer (mid‐August–September) as part of the Alaska Fisheries Science Centers' Bering Arctic Subarctic Integrated Survey (BASIS), 2002–2012, 2014, 2016, 2018 (Figure 3) (Alaska Fisheries Science Center, 2021). Data are available via the Alaska Ocean Observing System website (https://portal.aoos.org/#module‐metadata/d4fe79aa‐75b6‐11e4‐956f‐00265529168c). Stations (*n* = 1063) were approximately 30 to 60 nautical miles apart. A Cantrawl model 400/601 rope trawl net was towed from a vessel at 3.5–5 knots (6.5–9.3 km/h) for approximately 30 min. Tows were made during daylight hours. The sampling effort was quantified as the area swept by the net at each station. Area swept was estimated as the product of horizontal net opening (55 m on average) and distance towed. The distance towed was calculated as the haversine distance from the position of equilibrium (net deemed to be open and fishing) to haulback (the initial retrieval of the net). All fish caught were sorted, counted and weighed (kg) by species at each station. We used total catch in weight (kg) for juvenile sockeye and pink salmon and catch in numbers for age‐0 pollock because recording catch in weight for age‐0 pollock was initiated in 2003.

Sea temperatures were sampled at each station from surface to 5–10 m off bottom using a Seabird Electronics Inc. model 9 or 25 CTD. For our sea temperature estimate, we used values at 20 m depth (Temp_20m), the approximate mean vertical distribution of juvenile sockeye salmon (Manzer, 1964). Temperature at five stations was derived from heat maps of temperature in order to fill in missing observations to match survey locations using linear interpolation with the akima package version 0.6‐2 (Akima et al., 2016) in R (R Core Team, 2023).

Zooplankton were collected in the water column using bongo net tows at each station and analyzed using methods described in Coyle et al. (2011) and Eisner, Zuenko, et al. (2020). Although euphausiids are an important prey item of juvenile sockeye salmon, current collection methods were not adequate to quantify euphausiid abundance, so we chose to analyze *Calanus* copepod densities (#·m^−2^, hereafter referred to as densities), a less important zooplankton prey item of juvenile sockeye salmon. In addition, we analyzed densities of juvenile sockeye salmon in relation to age‐0 pollock, an important prey item of juvenile sockeye salmon in the EBS. In 2002–2011, zooplankton samples were collected with a 60‐cm bongo frame with 505 μm mesh. In 2012–2018, zooplankton samples were collected with both a 20‐cm bongo frame with 153 μm mesh nets and a 60‐cm frame with 505 μm mesh nets. *Calanus* counts were not significantly impacted by method changes (Kimmel & Duffy‐Anderson, 2020). Volume filtered was measured with a calibrated General Oceanics flowmeter located in the net opening. All zooplankton samples were preserved in 5% formalin buffered with 2.5% sodium borate and filtered seawater. The count of *Calanus* in a sample was calculated as the sum of copepodite stages III adult for *Calanus*. We derived the water column‐integrated values of *Calanus* (#·m^−2^) at each station by multiplying the mean abundance (#·m^−3^) by water column depth minus 10 m, the distance bongo nets were deployed off bottom at each station.

To examine the relative importance of prey items during late summer, stomachs were collected from juvenile sockeye salmon and contents analyzed for food habits, 2003 to 2018, except 2013 and 2017. At each sampling station, stomach contents from multiple fish were pooled and gut contents were sorted by taxa according to Farley Jr et al. (2005). Prey items were identified to the lowest possible taxonomic group on board the vessel (Davis et al., 2009). A stomach content index (SCI) for each taxa was calculated as the prey weight divided by the predator weight multiplied by 10,000 for the pooled samples at each station. We averaged the SCI for each prey category by year. Proportions of prey category were calculated and reported by year. The total number of stomachs included in this study was 2871 from 381 stations (Table A1). Broad prey categories were defined to capture the majority of the variation in juvenile sockeye salmon diet over our sample period (Table A2).

### 2.3 Statistical analysis

The Vector Autoregressive Spatio‐Temporal (VAST) modeling approach by Thorson et al. (2015) was used to (1) estimate and examine spatiotemporally varying patterns in juvenile sockeye salmon densities; (2) estimate annual indices of juvenile sockeye salmon distribution and abundance and covariates; and (3) estimate fixed effects of covariates on spatio‐temporally varying densities of juvenile sockeye salmon. The VAST model‐based approach to abundance estimation helps reduce bias in abundance estimates resulting from spatially unbalanced sampling across years, while propagating uncertainty resulting from predicting density in unsampled areas (Shelton et al., 2014). The model included a stochastic partial differential equation approximation to spatial and spatio‐temporal variables, which involved specifying a triangulated mesh among points in the VAST model. Spatio‐temporal models were generated using the VAST package version 3.10.0, INLA version 22.04.16, TMB version 1.9.1, FishStatsUtils version 2.12.0, R software version 4.11.3, and RStudio version 2022.02.3 (R Core Team, 2023; RStudio Team, 2022). See Thorson et al. (2015) for additional information on model structure.

### 2.4 Spatio‐temporal patterns in juvenile sockeye salmon densities

The VAST model was used to estimate juvenile sockeye salmon densities $d\left(s,t\right)$ and annual indices of salmon distribution and biomass, and covariates. The VAST model includes two linear predictors. The first linear predictors $p_{1}\left(i\right)$ is the predicted numerical density, affected both by encounter probability and catch rates. The second linear predictor (r) is residual variation in catch rates. We specify a Poisson‐link delta model for the probability of encounter and a gamma distribution to model positive catch rates. These first two equations are also the first two stages of estimating annual indices and the basis for examining the covariate effects on densities. Each predictor includes an intercept for the fixed effects of the year, and random effects describing spatial and spatio‐temporal variation. The first linear predictor $p_{1}\left(i\right)$ representing variation in log number density and the second linear predictor for average log biomass per group $p_{2}\left(i\right)$, for sample *i* are given in Equation 1.

(1) $$p_{1}\left(i\right)=\alpha_{t}+\sum_{f=1}^{n_{\omega 1}}\omega_{1}\left(s_{i}\;\right)+\sum_{f=1}^{n_{\varepsilon 1}}\varepsilon_{1}\left(s_{i},t_{i}\right)$$

$$p_{2}\left(i\right)=\alpha_{t}+\sum_{f=1}^{n_{\omega 2}}\omega_{2}\left(s_{i}\;\right)+\sum_{f=1}^{n_{\varepsilon 2}}\varepsilon_{2\left(s_{i},t_{i}\right)}$$

where $a_{t}$ is the fixed year effect, $\sum_{f=1}^{n_{\omega 1}}\omega_{1}\left(s_{i}\;\right)$ and $\sum_{f=1}^{n_{\omega 2}}\omega_{2}\left(s_{i}\;\right)$ are the spatial effects, and $\sum_{f=1}^{n_{\varepsilon 1}}\varepsilon_{1}\left(s_{i},t_{i}\right)$ and $\sum_{f=1}^{n_{\varepsilon 2}}\varepsilon_{2}\left(s_{i},t_{i}\right)$ are the spatio‐temporal effects within the VAST model. Symbols include $s_{i}$ for knot location, $t_{i}$ is year, and *i* is sample or station. Parameters include a spatial effect as omega (ω) and spatio‐temporal effect as epsilon $\left(\varepsilon \right)$. Appropriate link functions for the Poisson‐link delta model (Thorson, 2019b) are used to calculate encounter probability $r_{1}\left(i\right)$ and positive catch rate $r_{2}\left(i\right)$, as shown in Equation 2.

(2) $$r_{1}\left(i\right)=1-\exp\left(-a_{i}\times \exp\left(p_{1}\left(i\right)\right)\right)$$

$$r_{2}\left(i\right)=\frac{a_{i}\times \exp\left(p_{1}\left(i\right)\right)}{r_{1}\left(i\right)}\times \exp\left(p_{2}\left(i\right)\right)$$

This predicted biomass density $d\left(s,t\right)$ at each spatial location s and year t is the product of the encounter probability and positive catch rate given in Equation 3.

(3) $$d\left(s,t\right)=r_{1}\left(s,t\right)\times r_{2}\left(s,t\right)$$

### 2.5 Temporal trends and correlations among annual indices of sockeye biomass and distribution and covariates

Annual indices $I\left(t\right)$ of sockeye biomass, age‐0 pollock abundance, juvenile pink salmon biomass, total zooplankton, and mean Temp_20m by year t were estimated by summing the predicted density $d\left(s,t\right)$ values adjusted for area swept $a\left(s\right)$ over the entire survey area (Thorson, Pinsky, & Ward, 2016; Thorson, Rindorf, et al., 2016) given in Equation 4.

(4) $$I\left(t\right)=\sum_{s-}^{n_{s}}a\left(s\right)\times d\left(s,t\right)$$

where $a\left(s\right)$ is the area swept for a given location s, $d\left(s,t\right)$ is the predicted density at each location s and year t, and $n_{s}$ is the total number of discrete locations in space. Area swept was set as the product of the distance towed and horizontal opening (km^2^) of the trawl net for fish, 1 for temperature, and 0.0001 for *Calanus*, approximately equal to the radius of the bongo net.

The center of gravity, or distribution, ($z\left(t,m\right)$) of juvenile sockeye salmon by measure m and year t is given in Equation 5 (Thorson, Pinsky, & Ward, 2016; Thorson, Rindorf, et al., 2016).

(5) $$z\left(t,m\right)=\sum_{s-}^{n_{s}}\frac{z\left(s,m\right)\times a\left(s\right)\times d\left(s,t\right)}{I\left(t\right)}$$

where $z\left(s,m\right)$ is the center of gravity for each location s, $a\left(s\right)$ is the area swept at each location s, $d\left(t,s\right)$ is the predicted density for the location s in year t, and $I\left(t\right)$ is the biomass index for year t.

The effective area occupied $A\left(t\right)$ for each year t is estimated as the ratio of biomass $I\left(t\right)$ to average density $D\left(t\right)$ given in Equation 6 (Thorson, Pinsky, & Ward, 2016; Thorson, Rindorf, et al., 2016).

(6) $$A\left(t\right)=\frac{I\left(t\right)}{D\left(t\right)}$$

Time series of annual estimates and standard errors from VAST were plotted by year for each index. A second‐order polynomial regression model was used to describe the relationship between annual indices of juvenile sockeye salmon distribution and abundance and the covariates at α = 0.05.

### 2.6 Covariate effects on spatio‐temporal juvenile salmon densities

Next, juvenile sockeye salmon densities as the response variable were estimated with individual covariates specified as linear or quadratic effects using the VAST model. We build on Equations 1 and 2 by adding a term for the covariate given in Equation 7.

(7) $$p_{1}\left(i\right)=\alpha_{t}+\sum_{f=1}^{n_{\omega 1}}\omega_{1}\left(s_{i}\right)+\sum_{f=1}^{n_{\varepsilon 1}}\varepsilon_{1}\left(s_{i},t_{i}\right)+\sum_{p=1}^{n_{p}}\gamma_{1}\left(t_{i},p\right)X\left(x_{i},t_{i},p\right)$$

where $a_{t}$ is the fixed year effect, $\sum_{f=1}^{n_{\omega 1}}\omega_{1}\left(s_{i}\;\right)$ is the spatial effect, $\sum_{f=1}^{n_{\varepsilon 1}}\varepsilon_{1}\left(s_{i},t_{i}\right)$ is the spatio‐temporal effect, and $\sum_{p=1}^{n_{p}}\gamma_{1}\left(\;t_{i},p\right)X\left(x_{i},t_{i},p\right)$ is the nonlinear effect of density p covariates. Symbols include $s_{i}$ for knot location, $t_{i}$ is year, and *i* is sample or station, X is the covariate (Temp_20m, *Calanus*, juvenile pink salmon, and age‐0 pollock) from each sampling location. Parameters include a spatial effect as omega (ω), spatio‐temporal effect as epsilon $\left(\varepsilon \right)$, and covariate effect as gamma (γ). We used the natural log of covariates (*Calanus*, age‐0 pollock and juvenile pink salmon) plus one to linearize and normalize the distribution of the data due to the large number of zero catches. Each covariates' effect was estimated in a separate model, in part due to possible multicollinearity. Nine models included a spatio‐temporal model and eight spatio‐temporal models with four covariates specified as linear or nonlinear. We acknowledge that fitting multiple models may result in spurious relationships by chance alone.

The second linear predictor is given in Equation 8.

(8) $$p_{2}\left(i\right)=\alpha_{t}+\sum_{f=1}^{n_{\omega 2}}\omega_{2}\left(s_{i}\right)+\sum_{f=1}^{n_{\varepsilon 2}}\varepsilon_{2}\left(s_{i},t_{i}\right)+\sum_{p=1}^{n_{p}}\gamma_{2}\left(t_{i},p\right)X\left(x_{i},t_{i},p\right)$$

Predicted densities $d\left(s,t\right)$ were calculated for each location s and year t. Covariates were modeled as nonlinear effects to specify a B‐spline with a maximum of two degrees of freedom using the *bs* function from the *splines* R package (R Core Team, 2023).

Specifications of our VAST models included: (1) 500 “knots” for the grid, where the location of these knots was identified using a k‐means algorithm based on the location of survey observations across different years; (2) a 25 km extrapolation area from the center of each knot, which then allows for overlap in space among regions around knots; and (3) the epsilon bias‐correction estimator, in order to estimate annual values of index to account for retransformation bias when calculating derived quantities of abundance as a nonlinear function of random effects or high variance in random effects (Thorson & Kristensen, 2016). Model convergence requires that parameters are not within bounds and that the maximum absolute gradient of the log‐marginal‐likelihood must be close to zero.

Model performance was examined with predicted encounter probability quantiles and observed quantiles, quantile plots for residuals of the positive densities, and spatial trends in the Pearson residuals for encounter probability and positive catch rate components by knot and year. Cross‐validation with a simple random design was used to assess model predictions and observations for the full sample and threefold partitions of the data. Linear regression relationships were presented for the observed and predicted values of the full and partitioned datasets.

Percent deviance explained in the spatio‐temporal variation in densities of juvenile sockeye salmon by the addition of the covariate term in the VAST model is given in Equation 9.

(9) $$\%\text{Deviance Explained}=\frac{1-{\text{Deviance}}_{\text{Spatio}-\text{temporal with covariates}}}{{\text{Deviance}}_{\text{Spatio}-\text{temporal}}}$$

The percent deviance is the percent change in the spatio‐temporal variance (Epsilon term squared) between the 1st linear predictors of two models (Thorson, 2019a).

Density covariate effect plots were used to visualize the relationship between covariates and the 1st linear predictor. Maps showed the partial effect of modeled covariates, calculated as the product of covariates at each location and the estimated covariate response and then summing across covariates. A location with a coefficient of 0.1 indicates an approximately 10% increase in the predicted density at that location, with a resulting increase in both encounter probability and expected catch given an encounter.

## 3 RESULTS

### 3.1 Spatio‐temporal patterns in juvenile sockeye salmon densities

**FIGURE 4:**
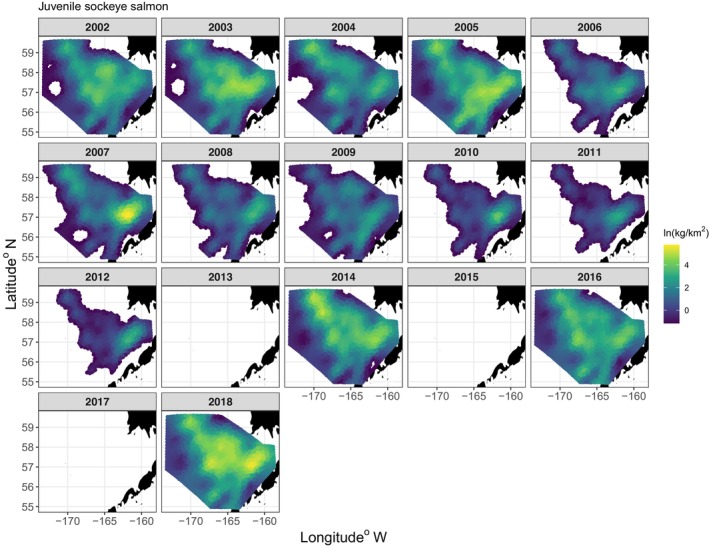
VAST estimated densities of juvenile sockeye salmon sampled in the southeastern Bering Sea during late summer, 2002–2012, 2014, 2016, and 2018. Yellow indicates high densities and blue indicates low densities. Light blue color indicates the extrapolation area.

**FIGURE 5:**
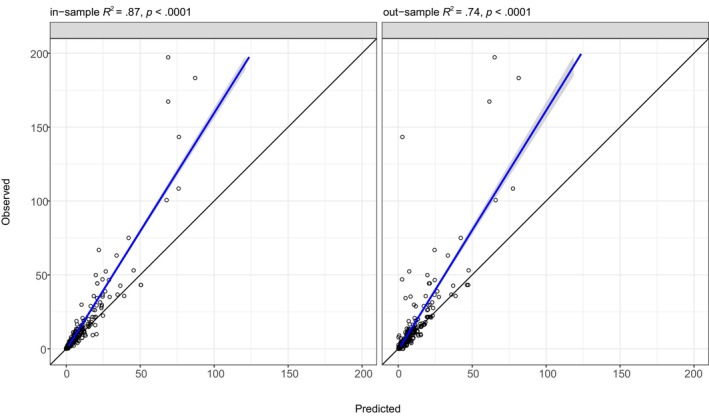
Linear regression model (blue line) and standard errors (gray band) relating the observed and predicted estimates of density of juvenile sockeye salmon sampled in surface waters (top 20 m) of the southeastern Bering Sea during late summer for the in‐ and out‐samples. The black line is the 1:1 replacement line.

Spatio‐temporal plots of the VAST estimated densities indicated that juvenile sockeye salmon distributed from southeast to northwest over the EBS shelf and had higher densities in the middle and inner domains in the east near Bristol Bay (Figure 4). The VAST juvenile sockeye salmon model without covariates had statistically significant spatial and spatio‐temporal variation in densities (Table A3). Model validation statistics indicate that the VAST model performed well and explained 87% of the variation in observed densities for the in‐sample (Figure 5). The out‐sample observed to predicted relationship had a slope similar to the in‐sample and explained 74% of the variability in densities. The VAST model‐based estimates of juvenile sockeye salmon densities did tend to underestimate observations at higher densities of juvenile sockeye salmon.

**FIGURE 6:**
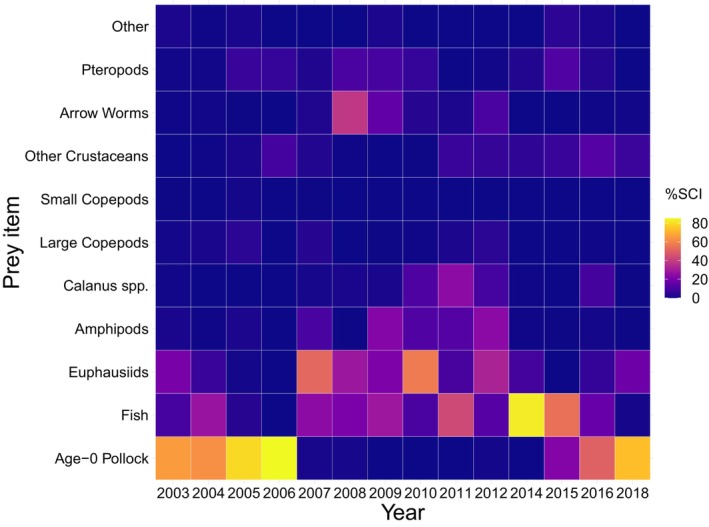
Diet proportions of juvenile sockeye salmon given as a stomach content index (%SCI) in the southeastern Bering Sea during late summer.

The prey items of juvenile sockeye salmon were primarily represented by age‐0 pollock, other fish, and euphausiids (Figure 6). In order of relative importance as indicated by the sum of the annual means of SCIs by group, prey categories include age‐0 pollock, other fishes, euphausiids, arrow worms, amphipods, pteropods, crustaceans, *Calanus* spp., large copepods, other taxa, and small copepods (Figure 6). During the warm stanza years, juvenile sockeye salmon fed primarily on age‐0 pollock, except for not feeding on age‐0 pollock during the 2014 warm year. During the cool stanza, juvenile sockeye salmon primarily fed on euphausiids, amphipods, *Calanus*, and other fish except for feeding on age‐0 pollock during 2006.

### 3.2 Hypothesis 1: Nonlinear effects of temperature on juvenile sockeye salmon

**FIGURE 7:**
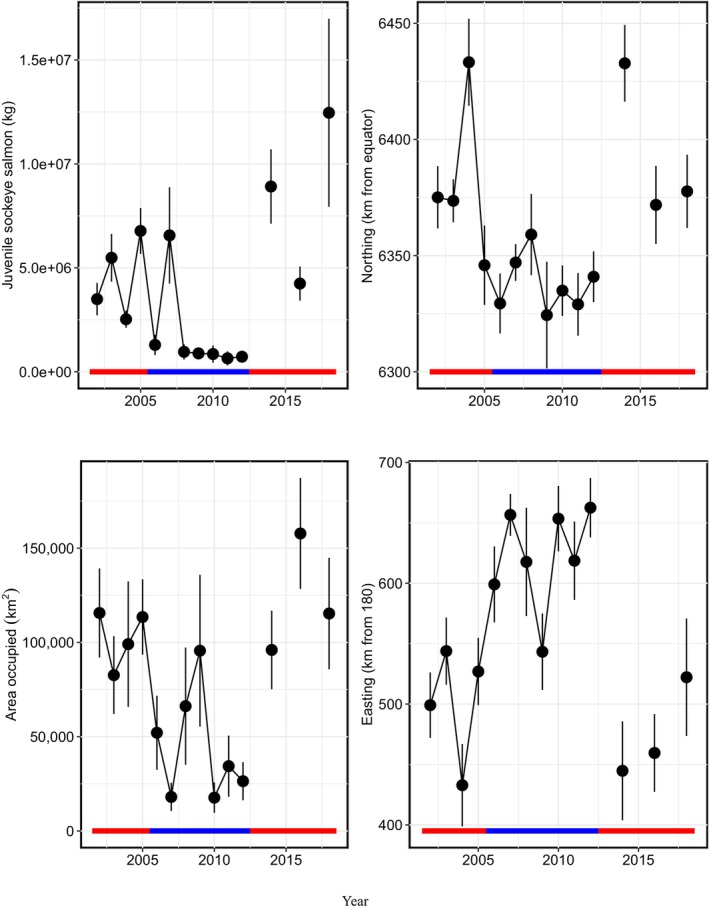
Time series of VAST means and standard errors of the annual estimates of juvenile sockeye salmon biomass (kg), northing (km from Equator), area occupied (km^2^) and easting (km from 180) in the southeastern Bering Sea during late summer, 2002–2012, 2014, 2016, and 2018. The horizontal line indicates red for warm years and blue for cold years.

**FIGURE 8:**
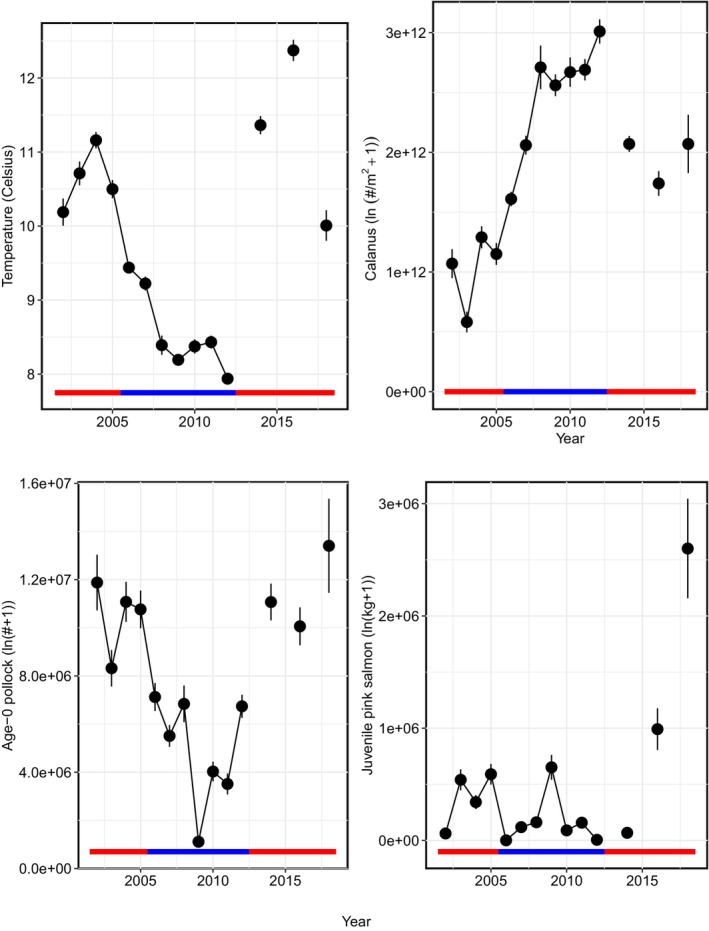
Time series of VAST means and standard errors of the annual estimates of sea temperature (°C) at 20 m depth, *Calanus* densities, age‐0 pollock abundance, and juvenile pink salmon biomass in the southeastern Bering Sea during late summer, 2002–2012, 2014, 2016, and 2018. The horizontal line indicates red for warm years and blue for cold years.

**FIGURE 9:**
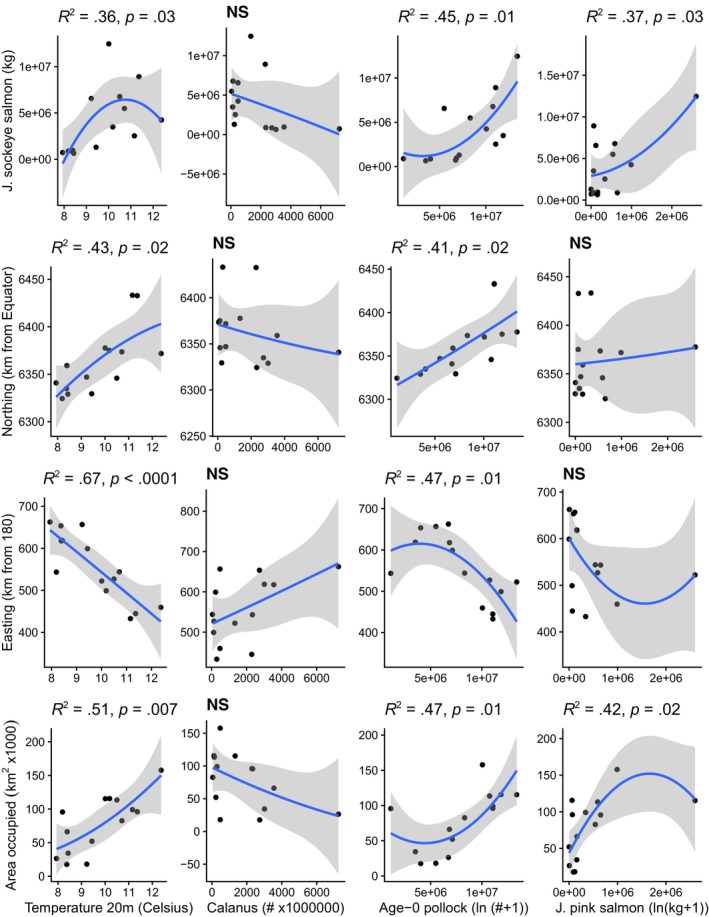
Polynomial regression models relating annual values of VAST estimates of juvenile sockeye salmon abundance, northing, easting, effective area occupied (EAO) and sea temperature (°C) at 20 m depth, *Calanus* densities, age‐0 pollock abundance, and juvenile pink salmon biomass.

Temporally, in part according to Hypothesis 1, total annual biomass of juvenile sockeye salmon had a significant nonlinear relationship with annual mean Temp_20m, with peak biomass occurring at 11°C (Figures 7, 8, 9). Northing and area occupied by juvenile sockeye salmon had a positive linear relationship with Temp_20m, while easting was negatively related to Temp_20m (Figure 9). Temporal trends in annual biomass and distribution (northing, easting, area occupied) of juvenile sockeye salmon indicate patterns related to warm and cool stanzas (Figure 7). Juvenile sockeye salmon had higher and more interannual variation in biomass during warm stanzas (2002–2005, 2014, 2016, and 2018) and lower and less interannual variation in biomass during the cool stanza (2006–2012), except for high biomass during 2007 (Figures 4 and 7). Temp_20m indicated a relatively warm stanza for years 2002–2005, a cool stanza for years 2006–2012, a warm stanza for years 2014–2018, and the warmest year during 2016 (Figure 8). Mean annual Temp_20m estimates ranged from 8.0 to 12.6°C from the VAST model and 8.0 to 12.3°C from design‐based means of the observed data. These temperatures were within the range of the preferred thermal preferences of juvenile sockeye salmon.

**FIGURE 10:**
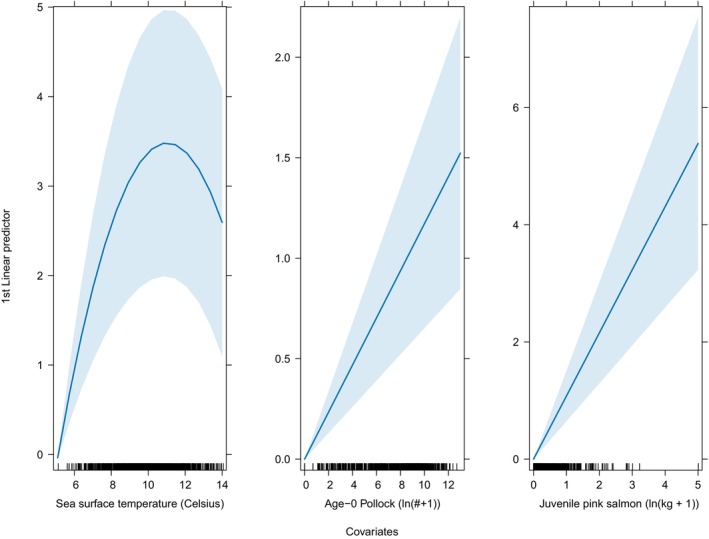
Effects of covariates on the log‐densities of juvenile sockeye salmon, arising both from increased encounter probability and higher density given an encounter model in the VAST model. Blue line is the model estimate. Light blue band is the 95% confidence interval.

**FIGURE A1:**
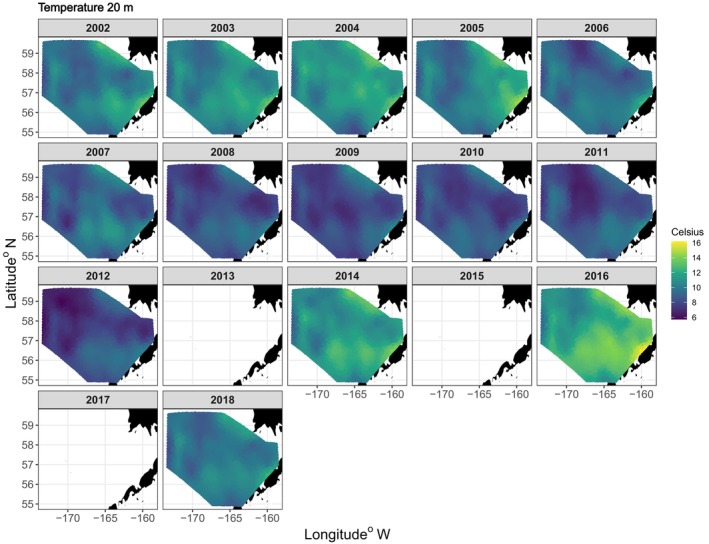
VAST predicted values of sea surface temperatures (at 20 m depth) at each station during the BASIS survey in the southeastern Bering Sea during late summer, 2002–2012, 2014, 2016, and 2018.

**FIGURE A2:**
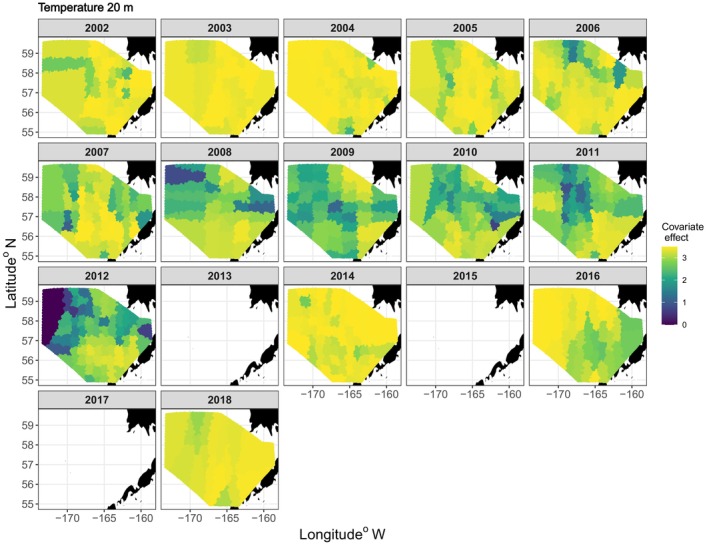
Partial effect of sea surface temperature on the 1st linear predictor of the Poisson‐linked delta model for predicting densities of juvenile sockeye salmon sampled in surface waters (top 20 m) of the southeastern Bering Sea during late summer, 2002–2012, 2014, 2016, and 2018, estimated with VAST. A location with a coefficient of 0.1 indicates an approximately 10% increase in the predicted density.

In the context of distribution, according to Hypothesis 1, a nonlinear effect of temperatures occurred on the spatio‐temporally varying densities of juvenile sockeye salmon (Figures A2 and 9). Temp_20m explained an additional 35% of the spatio‐temporal variation in the densities relative to the spatio‐temporal model alone (Table A3). Plots of densities of juvenile sockeye salmon indicated higher densities and a broader spatial distribution during warm years and lower densities with a more concentrated spatial distribution in the southeast middle domain near the Aleutian Islands during cool years (Figures 4 and A1). Among all years, station level observations of Temp_20m ranged from 5 to 14°C during the BASIS survey, whereas VAST estimates ranged from 6 to 16°C (Figure A1), within, below, and above the range of temperature preferences for juvenile sockeye salmon. Juvenile sockeye salmon are distributed primarily in waters between 8 and 14°C, with peak densities occurring at 11°C (Figures 9 and 10). Effects of Temp_20m on juvenile sockeye salmon densities were more widely spread during warm years than cold years, except for during the 2016 warm year (Figure A2).

### 3.3 Hypothesis 2: Positive effects of Calanus copepods on juvenile sockeye salmon

**FIGURE A3:**
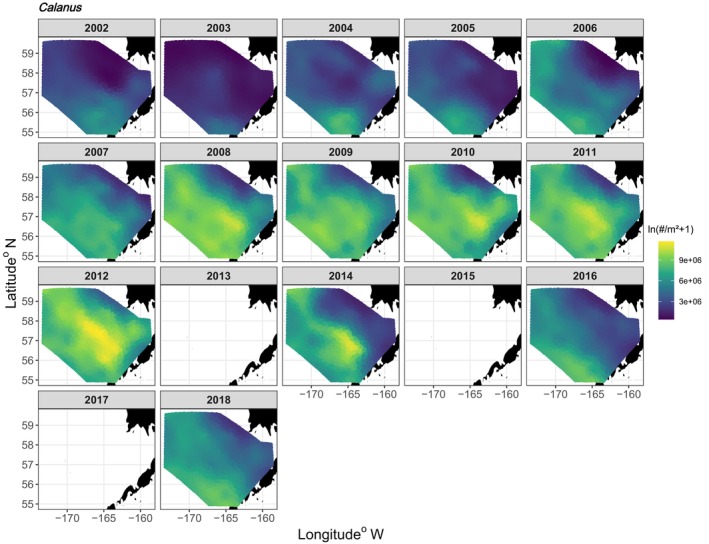
VAST predicted natural log of *Calanus* densities at each station during the BASIS survey in the southeastern Bering Sea during late summer, 2002–2012, 2014, 2016, and 2018. Warmer colors (yellow) indicate higher densities and cooler colors (blue) indicate lower densities.

Contrary to Hypothesis 2, the annual indices of biomass, northing, easting, and area occupied by juvenile sockeye salmon had no significant relationship with the total annual abundance of *Calanus* (Figures 7, 8, 9). Densities of *Calanus* were low during the first warm stanza (2002–2005) and the early part of the cool stanza (2006–2007), higher during the cool stanza (2008–2012) and the early part of the second warm stanza in 2014, and followed by lower densities during the latter part of the second warm stanza (2016, 2018) (Figure 8). The distribution of *Calanus* densities was patchy and less broadly distributed during the warm stanzas, except for during 2014 in the early part of the second warm stanza (Figure A3). *Calanus* densities had no significant effect on spatio‐temporal variation in juvenile sockeye salmon densities.

### 3.4 Hypothesis 3: Positive effects of age‐0 pollock on juvenile sockeye salmon

Temporally, consistent with Hypothesis 3, annual biomass, northing, and effective area occupied of juvenile sockeye salmon had a significant positive relationship with total annual abundance of age‐0 pollock, while easting was negatively related to age‐0 pollock abundance (Figures 7, 8, 9). The abundance of age‐0 pollock, a major prey item of juvenile sockeye salmon, was generally higher during warm stanzas (2002–2005, 2014, 2016, and 2018) and lower during the cool stanza (2006–2012) (Figure 8).

**FIGURE A4:**
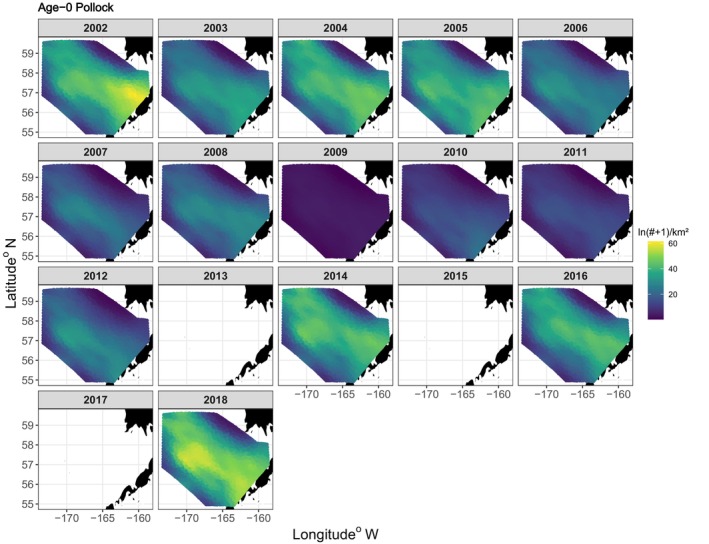
VAST predicted natural log of age‐0 pollock abundance plus one at each station during the BASIS survey in the southeastern Bering Sea during late summer, 2002–2012, 2014, 2016, and 2018.

**FIGURE A5:**
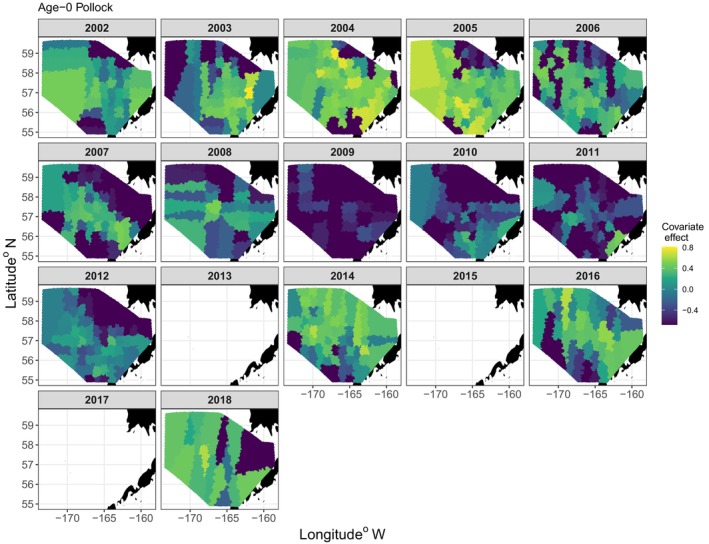
Partial effects of age‐0 pollock on the 1st linear predictor of the Poisson‐linked delta model predicting densities of juvenile sockeye salmon sampled in surface waters (top 20 m) of the southeastern Bering Sea during late summer, 2002–2012, 2014, 2016, and 2018, estimated with VAST. A location with a coefficient of 0.1 indicates an approximately 10% increase in the predicted density. Warm colors (yellow) indicate a positive effect and cooler colors (blue) indicate a weak effect.

Spatio‐temporally, consistent with our hypothesis, a positive relationship occurred between age‐0 pollock abundance and spatio‐temporally varying densities of juvenile sockeye salmon. The VAST estimates of age‐0 pollock show higher densities and a broader distribution during the warm stanzas relative to years during the cool stanza (Figure A4). Age‐0 pollock abundance explained an additional 15% of the spatio‐temporal variation in the densities of juvenile sockeye salmon relative to the spatio‐temporal model alone (Table A3). The covariate effects plot indicates an association between age 0‐pollock and juvenile sockeye salmon during warm stanzas (Figure A5).

### 3.5 Hypothesis 4: Negative effects of juvenile pink salmon on juvenile sockeye salmon

Temporally, opposite to Hypothesis 4, a positive relationship was found between juvenile pink salmon biomass and the biomass and area occupied by juvenile sockeye salmon, but juvenile pink salmon biomass did not relate significantly to the northing or easting of juvenile sockeye salmon (Figures 7, 8, 9). The annual biomass of juvenile pink salmon was much lower than the abundances of both juvenile sockeye salmon and age‐0 pollock (Figures 7 and 8). Juvenile pink salmon biomass was high during most warm stanza years (2003–2005, 2016, and 2018) except during 2002 and 2014, high in the 2009 cool year, and low in other cool years.

**FIGURE A6:**
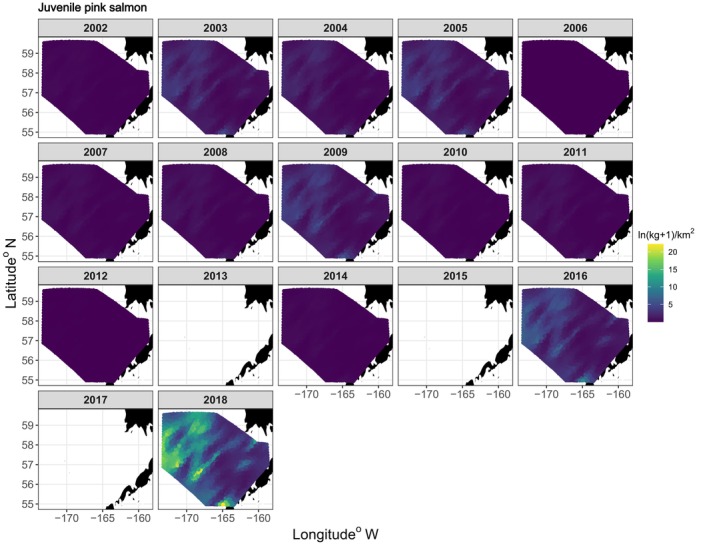
VAST predicted natural log of juvenile pink salmon biomass plus one at each station during the BASIS survey in the southeastern Bering Sea during late summer, 2002–2012, 2014, 2016, and 2018. Warmer colors (yellow) indicate higher densities and cooler colors (blue) indicate lower densities.

**FIGURE A7:**
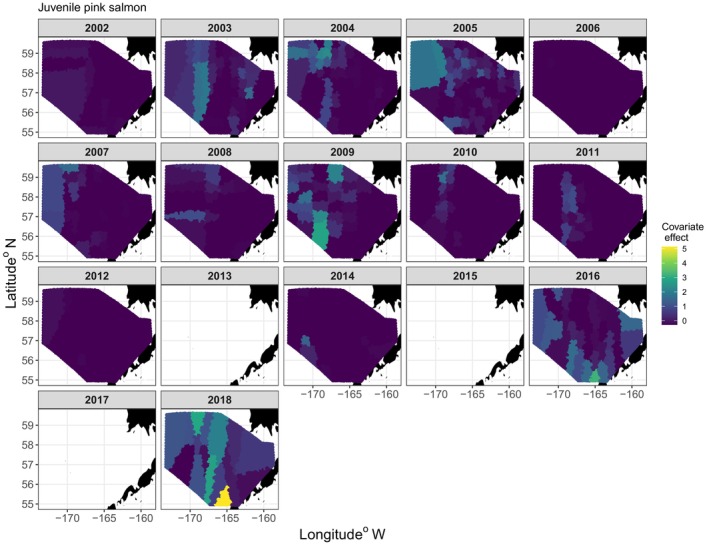
Partial effects of juvenile pink salmon on 1st linear predictor of the Poisson‐linked delta model predicting densities of juvenile sockeye salmon sampled in surface waters (top 20 m) of the southeastern Bering Sea during late summer, 2002–2012, 2014, 2016, and 2018, estimated with VAST. A location with a coefficient of 0.1 indicates an approximately 10% increase in the predicted density. Warm colors (yellow) indicate a positive effect and cooler colors (blue) indicate a weak effect.

Spatio‐temporally, juvenile pink salmon biomass had a positive linear effect on juvenile sockeye salmon densities and explained an additional 25% of the spatio‐temporal variation in the densities of juvenile sockeye salmon relative to the spatio‐temporal model alone (Table A3; Figure 10). The covariate effects plot indicates a stronger covariation between juvenile sockeye salmon and juvenile pink salmon during 2003–2005, 2009, 2016, and 2018. In particular, during 2018, the large densities of juvenile pink salmon located near the Alaska Peninsula corresponded with high densities of juvenile sockeye salmon in the area (Figures A6 and A7).

## 4 DISCUSSION

The world's largest run of sockeye salmon originates from the Bristol Bay river systems in Alaska. After spending several years in freshwater, these sockeye salmon rear as juveniles during the first year at sea in the marine waters of the EBS and as immatures and maturing (adults) sockeye salmon migrating to and from the Bering Sea and North Pacific Ocean. During recent warm years, these sockeye salmon have experienced record returns to the rivers as adults. Since the early 2000s, climate variation in the EBS has had major impacts on the marine ecosystem and trophic ecology of zooplankton and fish, favoring some species but not others. Salmon rely heavily on freshwater and early marine environments as juveniles for their survival. Understanding how species distribution, abundance, and marine habitat associations have varied in response to past climate variation, prey resources, and competitors can improve our understanding of how species may respond to future changes in marine ecosystems.

First, we explored biological and environmental factors affecting the annual indices of distribution and biomass of juvenile sockeye, and then we explored the effects of these factors on the intra‐annual or spatio‐temporally varying densities of juvenile sockeye salmon in the EBS (2002–2018). Specific mechanisms were proposed for covariates to affect our species of interest (Figure 2). Temporally, the annual biomass of juvenile sockeye salmon had a nonlinear association with the annual mean September sea temperature, a positive association with the total abundance of age‐0 pollock and the total biomass of juvenile pink salmon, and no significant relationship with *Calanus* densities. Based on our analyses of the fixed effects of covariates on spatio‐temporally varying densities of juvenile sockeye salmon, we detected a nonlinear effect of sea temperature, a positive association with age‐0 pollock abundance and juvenile pink salmon biomass, and no association with *Calanus*. Retrospective analyses indicate that variability in our biomass and density estimates of juvenile sockeye salmon was due to greater abundance (higher survival) or the same survival rates (i.e. higher spawner abundance) rather than greater body size (higher growth rates), as indicated by the positive and significant correlation among total catch in biomass and catch in numbers but not mean body weight among stations.

### 4.1 Hypothesis 1: Nonlinear effect of temperature on juvenile sockeye salmon

Consistent with our hypothesis, a nonlinear relationship was found between the annual mean September sea temperature and annual estimates of juvenile sockeye salmon biomass in the EBS, 2002–2018. The initial biomass increase is hypothesized to be due to warming and earlier spring ice break up in rivers, earlier timing of smolt migration from freshwater to saltwater, an increase in freshwater zooplankton densities, increases in early marine pelagic production, higher growth rate potential, higher energy reserves prior to winter, and increases in early marine body condition and body size (Dailey, 2020; Farley Jr, Murphy, Adkison, & Eisner, et al., 2007; Schindler et al., 2005). A reduction in biomass occurred in 2016, during an exceptionally warm year (>12°C). During 2016, temperatures were exceptionally warm due to a mass of warm water, called the “warm blob,” that moved into the EBS from the Gulf of Alaska (Stabeno et al., 2017). The warm sea temperatures during 2016 were associated with reduced body condition in age‐0 pollock in the Gulf of Alaska; however, positive and negative effects varied by species (Rogers et al., 2020; Suryan et al., 2021). As ectotherms, fish are highly sensitive to changes in water temperature that in turn influence their physiology, metabolism, and behavior (Cox, 1968). Based on our findings, we expect a reduction in the annual biomass of juvenile sockeye salmon at mean September temperatures of <10 and >12°C.

Similarly, we found a nonlinear effect of temperature on spatio‐temporally varying densities of juvenile sockeye salmon in the EBS, indicating a thermal preference in their distribution ranging from 8 to 14°C and peak densities at 11°C, within the range of 5–14°C in our study. In an earlier study from 1965 to 2009, juvenile sockeye salmon did not show preference for specific temperatures within the range of 7.3–14.6°C but did distribute with specific bottom depth and salinity in Alaskan waters of the EBS and Gulf of Alaska (Echave et al., 2012). Our findings suggest that sea temperatures at or above 12°C represent a temperature threshold that limits the densities of juvenile sockeye salmon, while sea temperatures around 11°C had the strongest positive effect on their densities.

With warming, juvenile sockeye salmon distributed further north and west and expanded their range over a broader region. Farley Jr, Murphy, Adkison, & Eisner, et al. (2007) found a similar pattern of offshore and northward distribution during warm years 2002–2003 relative to cooler years 2000–2001. During late summer, juvenile sockeye salmon distribute primarily in the middle domain of the southern EBS, with a pattern of moving from Bristol Bay to oceanic waters in the basin of the central Bering Sea and south near the Aleutian Chain. Higher densities in the northwest outer domain and south of the Pribilof Islands indicate a west and southerly migration from the shelf to oceanic waters around the Pribilof Islands and movement north in the middle domain. During 2002–2005, an extensive offshore distribution of juvenile sockeye salmon may be the result of warmer offshore sea‐surface temperatures during spring and summer (Farley Jr, Murphy, Adkison, & Eisner, et al., 2007; Farley Jr et al., 2005), where warmer sea temperatures offer opportunities for rapid offshore movement, possibly due in part to higher growth rates related to increased productivity on the EBS shelf (Farley Jr, Murphy, Adkison, & Eisner, et al., 2007). The presence of more juvenile sockeye salmon in the northern portion of our survey may also be due to the increased presence of Nushagak River sockeye salmon that originate farther north than Bristol Bay sockeye salmon (Seeb et al., 2011). Predicted climate effects on the distribution of many groundfish, fish, and crab species in the EBS indicate slight shifts primarily north, but south for several species (Rooper et al., 2021). Benthic species distribution is limited by a benthic “cold pool” (<2°C) in the EBS that forms during winter and remains during summer, whereas juvenile salmon reside in the pelagic waters and are less limited by benthic temperatures in their movement north. We hypothesize that these juvenile sockeye salmon, at the northern extent of their range, move north during warm years to seek optimal temperatures, find thermal refuge from predators above the cold pool, conserve energy at lower temperatures, and/or seek prey items (Duffy‐Anderson et al., 2017). Other factors that influence species shifts northward include size structure or unexplained spatio‐temporal variation (Thorson et al., 2017). The estimated shift in the distribution west and north and the expanded ranges of juvenile sockeye salmon have both positive and negative implications for growth, feeding, and survival. For juvenile sockeye salmon, shifts northward and expanded ranges may also expose them to alternative predators (i.e., bird colonies on St. Lawrence Island), fewer prey (i.e., fewer age‐0 pollock), and competitor communities (i.e., more herring). Little is known about the mechanism driving this change in distribution during warming; however, annual prey availability may be influencing their overall distribution and abundances.

### 4.2 Hypothesis 2: Positive effects of *Calanus* copepods on juvenile sockeye salmon

Contrary to our hypothesis, *Calanus* did not explain additional variation in the annual biomass and distribution indices or spatio‐temporally varying densities of juvenile sockeye salmon. Zooplankton often play an important role in providing high‐quality nutrients to small fish in the EBS. For example, the lipid‐rich large copepod *Calanus*, a high‐quality prey, is linked to increased energy density and survival of age‐0 pollock (Eisner, Yasumiishi, et al., 2020; Heintz et al., 2013). While *Calanus* play an important role in providing high‐quality prey for small fishes in the EBS (Eisner, Yasumiishi, et al., 2020; Farley Jr et al., 2016; Heintz et al., 2013), they are not a major prey item for juvenile sockeye salmon. Juvenile sockeye salmon generally consumes euphausiids and fish during cool years and age‐0 pollock during warm years. We note that *Calanus* were distributed in the center and southwestern regions of the survey area, whereas juvenile sockeye salmon were distributed farther east and north. Therefore, the lack of spatial association between the density of *Calanus* and the densities of juvenile sockeye salmon indicates that juvenile sockeye salmon do not rely heavily on *Calanus* as a prey item. Understanding spatio‐temporal overlap with other important prey items, such as euphausiids, would provide more insight into warming‐related factors driving changes in the distribution and abundance of juvenile sockeye salmon.

### 4.3 Hypothesis 3: Positive effects of age‐0 pollock on juvenile sockeye salmon

According to our hypothesis, we found a strong positive relationship between the annual abundances of age‐0 pollock and juvenile sockeye salmon in the EBS. Age‐0 pollock are a highly abundant and important prey item for juvenile sockeye salmon, especially during warm years. During warm years, age‐0 pollock are the most abundant forage fish in pelagic waters, followed by juvenile sockeye salmon (Yasumiishi et al., 2020); therefore, predation pressure from juvenile sockeye salmon is likely minimal. During warm years (2002–2003) relative to cool years (2000–2001), juvenile sockeye salmon not only consumed age‐0 pollock as their primary prey item but also had a higher body condition and a larger body size (Farley Jr, Murphy, Adkison, & Eisner, et al., 2007). Juvenile sockeye salmon also had higher growth rate potential during warm years, when prey densities were positively related to spring sea temperature in the EBS (Farley & Trudel, 2009). Similar mechanisms may be driving the production of these two species that rely on similar prey items, or perhaps age‐0 pollock as a prey item are driving the marine survival of juvenile sockeye salmon.

Spatio‐temporally, according to our hypothesis, age‐0 pollock had a positive association with the densities of juvenile sockeye salmon, especially during warm years. During both warm and cool years, juvenile sockeye salmon remain in the upper water column, while age‐0 pollock distribute at higher densities in the upper water column during warm years and deeper in the water column during cool years (Parker‐Stetter et al., 2013), making age‐0 pollock more accessible to juvenile sockeye salmon as a prey item during warm years. The latitudinal distribution of juvenile sockeye salmon was farther north and over a larger area in years with higher densities of age‐0 pollock. Our finding that juvenile sockeye salmon distribute with age‐0 pollock indicates that juvenile sockeye salmon distribution is potentially influenced by the distribution of major prey resources.

### 4.4 Hypothesis 4: Negative effects of juvenile pink salmon on juvenile sockeye salmon

Contrary to our hypothesis, a positive rather than negative association occurred between the annual biomass of juvenile sockeye salmon and juvenile pink salmon. This positive association may indicate a common driver in freshwater or the early marine environment for the survival of these two species of salmon. During 2017–2021, the abundance of Bristol Bay sockeye salmon and pink salmon in the region had improved returns, while other salmon species originating from western Alaska had negative or no trends in abundance (Munro, 2023). Further analysis of the quality and quantity of prey relative to spatio‐temporal variation in juvenile salmon densities and body condition would aid in understanding common drivers of survival. In addition, we found that the biomass of juvenile pink salmon was an order of magnitude lower than that of juvenile sockeye salmon, so competition for shared prey items was likely minimal.

Similarly, a positive effect of juvenile pink salmon on the spatio‐temporally varying densities of juvenile sockeye salmon suggests no significant competition for food or niche partitioning between these species. Intense interspecific competition can restrict or displace a niche and lead to habitat partitioning (Cox, 1968). The presence of competitors can lead to changes in the distribution and abundance of plants, birds, fish, and mammals (Cox, 1968). The magnitude of competition can also vary with dynamic temporal and spatial‐scale events such as glaciation, continental drift, seasonal migrations, and climate change (Cox, 1968; Mayr & Meise, 1930). Understanding spatio‐temporal variation in competitor densities provides insight into seasonal migration patterns of species used to maximize feeding, growth, and survival. For example, in the central Bering Sea, the highly abundant adult pink salmon can have significant density‐dependent effects on the distribution, feeding, growth, and survival of other adult salmon species (Ruggerone et al., 2003; Tadokoro et al., 1996). The potential for competition between juvenile pink and sockeye salmon on the EBS shelf stems from commonality in their prey, both fed primarily on euphausiids during cold years and age‐0 pollock during warm years (Farley et al., 2006). Juvenile pink and sockeye salmon may be cuing in the same spatial domain, where they may compete and incur poorer individual body conditions (Beamish et al., 2010). Additional analyses of competitor and prey densities are needed in relation to the body condition of juvenile sockeye salmon. However, results indicate that there is sufficient prey resource to support the densities of both species in the EBS during late summer.

### 4.5 Management implications

Identifying essential fish habitats provides a baseline for future conservation and management decisions (Magnuson‐Stevens Fishery Conservation and Management Act, 1976). These management decisions may include protecting habitats that fish use to spawn, feed, grow, and mature. The EBS is a major and essential habitat for the feeding, growth, and survival of juvenile sockeye salmon; however, many of these biological attributes are not mapped. In our study, we identified and mapped thermal and prey fields that impact spatio‐temporal variation in the densities of juvenile sockeye salmon in the EBS. Monitoring these essential fish habitats allows for the identification of potential future areas of concern with conditions of major ecological function, sensitive stressors, and rare habitats.

For management purposes, the results of this study can be used in the development of forecast models for the survival of juvenile sockeye salmon for use in the management of federal and state fisheries. For example, the estimated abundance of juvenile salmon is often a leading indicator for adult salmon returns, indicating that production is determined during freshwater and early marine life stages (Farley Jr et al., 2020; Murphy et al., 2017). Our results indicate that variations in sea temperature, juvenile pink salmon biomass, age‐0 pollock abundances, and annual abundance of juvenile sockeye salmon may be useful in models predicting future returns of adult sockeye salmon to Bristol Bay river systems. Due to the multiple populations and age structure of Bristol Bay sockeye salmon, future collections of scales and otoliths for age and tissues for genetic analysis by river would inform the stock structure of juvenile salmon captured at sea and help link the abundances of juvenile sockeye salmon to the returns of adult sockeye salmon to Bristol Bay. An evaluation of how the distribution and abundance of Pacific salmon have changed in response to past and present spatial and temporal ecosystem change will help us understand how Pacific salmon will respond to future climate warming. This improved understanding of the spatial and temporal changes in the ecology of juvenile salmon can inform climate‐adaptive fishery and spatial management policies.

## AUTHOR CONTRIBUTIONS

**Ellen M. Yasumiishi:** Conceptualization (lead); formal analysis (equal); supervision (equal); writing – original draft (lead); writing – review and editing (equal). **Curry J. Cunningham:** Conceptualization (equal); formal analysis (equal); methodology (lead); writing – review and editing (equal). **Ed V. Farley Jr.:** Conceptualization (equal); data curation (equal); investigation (equal); writing – review and editing (equal). **Lisa B. Eisner:** Conceptualization (equal); investigation (equal); writing – review and editing (equal). **Wesley W. Strasburger:** Conceptualization (equal); investigation (equal); writing – review and editing (equal). **John A. Dimond:** Data curation (equal); writing – review and editing (equal). **Paul Irvin:** Conceptualization (equal); visualization (equal).

## FUNDING INFORMATION

Funding for this work was provided by the National Marine Fisheries Service, the Arctic‐Yukon‐Kuskokwim Sustainable Salmon Initiative project numbers 45491 and 45557, North Pacific Research Board 285, BEST‐ Bering Sea Integrated Ecosystem Research Program 17, and the Alaska Department of Fish and Game. Cunningham is supported through the NOAA Quantitative Ecology and Socioeconomics Training (QUEST) Program and the Bristol Bay seafood processors.

## CONFLICT OF INTEREST STATEMENT

The authors have no conflict of interest to declare.

### OPEN RESEARCH BADGES

This article has earned Open Data and Open Materials badges. Data and materials are available at https://portal.aoos.org/#module‐metadata/d4fe79aa‐75b6‐11e4‐956f‐00265529168c.

## Data Availability

Data are available via the Alaska Ocean Observing System website (https://portal.aoos.org/#module‐metadata/d4fe79aa‐75b6‐11e4‐956f‐00265529168c).

## APPENDIX 1

**TABLE A1:** Number of stomach samples analyzed, fullness, and stomach content index (SCI) for juvenile sockeye salmon per station and year.

Year	No. stations	No. stomachs	Fullness	% Pollock SCI
2003	34	312	167	65
2004	77	629	142	63
2005	82	559	175	79
2006	4	7	118	85
2007	37	277	154	1
2008	13	74	183	1
2009	13	82	88	0
2010	15	77	172	0
2011	7	50	56	0
2012	13	93	133	1
2014	31	292	241	0
2015	13	34	222	23
2016	29	258	149	50
2018	13	127	130	73

**TABLE A2:** Prey taxa in the diets of juvenile sockeye salmon in the eastern Bering Sea during late summer.

Prey group: common and scientific names
Other taxa: Balanidae, *Beroe* spp., *Berryteuthis magister*, Cephalopoda, Cnidaria, Copepoda, Diptera, insects, Oikopleura spp., and unidentified organic contents
Pteropods: *Clione limacina*, *Clione* spp., *Limacina helicina*, *Limacina* spp.
Arrow worms: *Chaetognatha*, *Parasagitta elegans*
Other crustaceans: *Acanthomysis* spp., Bivalvia, Caridea, *Chionoecetes opilio*, *Chinoecetes* spp.
Small copepods (*Centropages abdominalis*, *Eurytemora* spp., *Oncaea* spp., *Pseudocalanus* spp., *Tortanus discaudatus*)
Large copepods: *Epilabidocera amphitrites*, *Eucalanus bungii*, *Metridia pacifica*, *Neocalanus cristatus*
Calanus spp.: *Calanus glacialis*, *Calanus marshallae*
Amphipods: *Cyphocaris* spp., Gammaridae, *Hyperia medusarum*, *Hyperia* spp., *Hyperiidae*, *Hyperoche medusarum*, *Hyperoche* spp., *Themisto libellula*, *Themisto pacifica*
Euphausiids: Euphausiacea, Euphausiids, *Thysanoessa inermis*, *Thysanoessa inspinata*, *Thysanoessa longipes*, *Thysanoessa raschii*, Thysanoessa spp., Thysanoessa *spinifera*
Other fishes: *Ammodytes* spp., Cottidae, fish eggs, unidentified fish parts, Hexagrammidae, *Hexagramos stelleri*, *Mallotus villosus*, Pleuronectidae, *Sebastes* spp.
Age‐0 pollock: *Gadus chalcogrammus*

**TABLE A3:** VAST model output for the spatio‐temporal models and spatio‐temporal models with covariates.

Covariate	None			Temperature			Pink salmon			Age‐0 pollock		
Parameter	Estimate SE	*t*‐value		Estimate SE	*t*‐value		Estimate SE	*t*‐value		Estimate SE	*t*‐value	
ln_H_input	0.45	0.15	2.93	0.40	0.16	2.47	0.33	0.15	2.26	0.46	0.16	2.95
ln_H_input	0.18	0.18	1.00	0.24	0.20	1.18	0.28	0.17	1.61	0.20	0.19	1.09
beta_2002	1.60	0.80	1.99	−1.64	1.04	−1.57	1.80	0.86	2.10	1.62	0.70	2.32
beta_2003	1.28	0.78	1.64	−2.07	1.05	−1.98	1.24	0.84	1.47	1.39	0.67	2.06
beta_2004	1.42	0.76	1.86	−1.97	1.04	−1.90	1.45	0.82	1.77	1.26	0.65	1.93
beta_2005	2.40	0.79	3.02	−0.86	1.03	−0.83	2.22	0.85	2.61	2.27	0.70	3.26
beta_2006	−0.51	0.79	−0.65	−3.58	1.02	−3.51	−0.24	0.84	−0.29	−0.39	0.68	−0.57
beta_2007	−0.14	0.78	−0.17	−3.15	1.00	−3.14	−0.01	0.83	−0.01	0.18	0.67	0.27
beta_2008	0.28	0.80	0.35	−2.37	0.98	−2.43	0.39	0.86	0.45	0.46	0.70	0.65
beta_2009	1.07	0.77	1.39	−1.46	0.92	−1.58	0.99	0.83	1.19	1.81	0.68	2.65
beta_2010	−0.89	0.80	−1.11	−3.54	0.97	−3.64	−0.76	0.86	−0.88	−0.40	0.71	−0.56
beta_2011	−1.02	0.82	−1.24	−3.77	1.01	−3.75	−0.89	0.87	−1.02	−0.44	0.72	−0.62
beta_2012	−0.88	0.80	−1.09	−3.38	0.96	−3.50	−0.63	0.86	−0.73	−0.51	0.70	−0.73
beta_2013	3.01	0.78	3.87	−0.16	1.00	−0.16	2.93	0.84	3.50	2.87	0.68	4.25
beta_2014	1.22	0.77	1.58	−2.12	1.04	−2.04	1.45	0.83	1.75	1.22	0.66	1.84
beta_2015	3.50	0.81	4.30	0.29	1.03	0.29	3.37	0.87	3.88	3.35	0.71	4.74
beta_2016	2.70	0.86	3.15	−0.39	1.10	−0.35	2.54	0.92	2.77	2.86	0.77	3.73
beta_2017	3.45	0.81	4.26	0.23	1.03	0.22	3.28	0.87	3.78	3.31	0.71	4.69
beta_2018	2.00	0.85	2.34	−1.32	1.08	−1.22	1.29	0.93	1.39	1.97	0.75	2.64
gamma1_cp				5.28	1.19	4.42	0.52	0.11	4.91	0.45	0.10	4.42
gamma1_cp				2.56	0.77	3.34						
L_omega1_z	1.39	0.26	5.36	1.41	0.26	5.39	1.35	0.25	5.29	1.25	0.24	5.16
L_epsilon1_z	0.51	0.10	5.24	0.41	0.10	4.04	0.44	0.10	4.65	0.47	0.11	4.46
L_epsilon1_z^∧^2	0.26			0.17			0.20			0.22		
% change in L_epsilon1_z^∧^2				35%			25%			15%		
logkappa1	−4.52	0.17	−26.21	−4.47	0.19	−23.70	−4.64	0.18	−25.32	−4.45	0.18	−24.69
beta_2002	−0.95	0.59	−1.62	−0.74	0.91	−0.81	−1.01	0.62	−1.62	−0.90	0.62	−1.43
beta_2003	−0.55	0.57	−0.96	−0.32	0.92	−0.35	−0.69	0.61	−1.13	−0.61	0.61	−1.00
beta_2004	−0.97	0.52	−1.85	−0.73	0.90	−0.81	−1.11	0.56	−1.99	−0.76	0.56	−1.35
beta_2005	−1.04	0.58	−1.79	−0.84	0.89	−0.94	−1.01	0.60	−1.67	−0.85	0.62	−1.37
beta_2006	−0.53	0.61	−0.86	−0.42	0.92	−0.45	−0.64	0.65	−0.98	−0.56	0.65	−0.88
beta_2007	0.01	0.58	0.02	0.17	0.88	0.19	−0.06	0.61	−0.09	−0.22	0.62	−0.36
beta_2008	−0.95	0.60	−1.60	−0.92	0.83	−1.11	−1.05	0.63	−1.65	−1.15	0.63	−1.81
beta_2009	−1.38	0.54	−2.56	−1.41	0.77	−1.83	−1.48	0.58	−2.58	−1.95	0.60	−3.25
beta_2010	−0.77	0.62	−1.25	−0.71	0.82	−0.86	−0.86	0.65	−1.34	−1.07	0.65	−1.64
beta_2011	−0.69	0.66	−1.04	−0.63	0.91	−0.70	−0.84	0.69	−1.21	−1.19	0.70	−1.70
beta_2012	−1.19	0.67	−1.77	−1.16	0.90	−1.29	−1.32	0.70	−1.88	−1.42	0.70	−2.02
beta_2013	−2.12	0.56	−3.79	−2.06	0.87	−2.36	−2.12	0.59	−3.61	−1.96	0.60	−3.28
beta_2014	0.09	0.56	0.16	0.38	0.93	0.41	0.07	0.60	0.11	0.13	0.60	0.22
beta_2015	−2.55	0.61	−4.20	−2.49	0.91	−2.74	−2.59	0.64	−4.05	−2.39	0.64	−3.76
beta_2016	−1.26	0.64	−1.97	−1.01	0.98	−1.03	−1.41	0.69	−2.04	−1.39	0.67	−2.06
beta_2017	−2.46	0.60	−4.08	−2.38	0.91	−2.63	−2.50	0.64	−3.93	−2.30	0.63	−3.63
beta_2018	−0.09	0.67	−0.14	0.06	0.97	0.06	−0.48	0.74	−0.65	−0.03	0.69	−0.04
gamma2_cp				0.17	1.23	0.13	−0.04	0.11	−0.32	−0.41	0.11	−3.66
gamma2_cp				−0.57	0.80	−0.71						
L_omega2_z	1.39	0.17	8.10	1.36	0.17	8.15	1.34	0.17	7.96	1.46	0.18	8.10
L_epsilon2_z	0.82	0.08	9.81	0.78	0.09	8.93	0.84	0.08	10.69	0.82	0.09	9.48
L_epsilon2_z^∧^2	0.68			0.61			0.71			0.67		
% change in L_epsilon2_z^∧^2				11%			−4%		1%			
vs spatial model												
vs spatio‐temporal												
logkappa2	−3.98	0.15	−27.10	−3.93	0.15	−25.86	−4.09	0.14	−29.02	−4.03	0.14	−28.09
logSigmaM	−0.24	0.05	−4.60	−0.23	0.05	−4.33	−0.26	0.05	−5.23	−0.24	0.05	−4.56
AIC	3613			3592			3550			3594		

*Note*: Parameters include anisotropy (ln_H), year effect (beta), covariate effect (gamma), 1st linear predictor (1), 2nd linear predictor (2), loading matrix for the spatial variation (L_omega), loading matrix for the spatio‐temporal variation (L_epsilon), decorrelation distance (log kappa), and error distribution (logSigmaM). Summary statistics include the Akaike information criterion (AIC).
